# Multiple attribute decision making model and application to food safety risk evaluation

**DOI:** 10.1371/journal.pone.0189835

**Published:** 2017-12-19

**Authors:** Lihua Ma, Hong Chen, Huizhe Yan, Lifeng Yang, Lifeng Wu

**Affiliations:** 1 School of Management, China University of Mining and Technology, Xuzhou, P.R. China; 2 School of Management Engineering and Business, Hebei University of Engineering, Handan, P.R. China; 3 School of Economic, Fuyang Normal University, Fuyang, P.R. China; Jiangnan University, CHINA

## Abstract

Decision making for supermarket food purchase decisions are characterized by network relationships. This paper analyzed factors that influence supermarket food selection and proposes a supplier evaluation index system based on the whole process of food production. The author established the intuitive interval value fuzzy set evaluation model based on characteristics of the network relationship among decision makers, and validated for a multiple attribute decision making case study. Thus, the proposed model provides a reliable, accurate method for multiple attribute decision making.

## Introduction

Safe food production is a major public safety problem in China [1], and the choice of food supplier is directly related to the level of food safety risk [2]. Therefore, the selection of a food supplier is directly related to the risk level of food safety [3]. It can be known from the decision-making theory that the selection of food suppliers is also a multiple attribute decision making (MADM) problem in management science [4].

Data acquisition for many management research problems relies on questionnaire surveys. However, the objective world is complex and uncertain [5,6], and ensuring accurate and objective evaluation of behaviors at an interview is often difficult [7]. Zadeh (1965) proposed fuzzy set theory to evaluate uncertainty [8], but many subsequent studies have found that the hesitation of behavioral subjects to deal with specific problems remains an obstacle to discuss uncertainty. To address this, Atanassov (1986) proposed interval valued intuitive fuzzy sets (IVIFSs) [9], which provide more accurate expression of uncertain information [10]. Fuzzy information integration has been extensively applied to IVIFSs, and many integrated operators proposed, such as intuitive fuzzy weighted and intuitive fuzzy mixed mean operators [11–14]. Subsequently, improved IVIFSs have been widely used in multiple attribute decision making and market forecasting [15–17]. Based on different fuzzy preference relation and ideal interval value, the experts put forward intuitionistic fuzzy preference relation, and give the same location element analysis through the similarity degree of different decision maker[18]. Fan (2011) proposed interval value intuitive fuzzy sets for statistical judgment and decision making in the presence of fuzziness [19]. The interval number kernel and interval value intuitive fuzzy numbers are defined and operator properties and application of the aggregation operator in multiple attribute decision making are discussed [20].

At present, researches on supplier selection and evaluation methods based on food safety are rare, because the food industry is one of the special industries with high risk[1–2]. In the present study, the evaluation system of food safety risk has been established from the perspective of the whole process of food production and safety, according to the characteristics of network relation with multi-agent decision making and the specialty of purchase decision making in supermarket food. Based on this, the IVIFS model with network relation characteristics was introduced to carry out the evaluation on food safety risk, providing some realistic guidance for the management and control of food safety level. This paper proposes a food safety risk assessment system for supermarket food suppliers, explicitly considering food production. Food safety risk assessment is conducted using an IVIFS model, which provides practical guidance for food safety control.

## Evaluation index for food safety risk

It can be seen from literature reviews that food safety evaluation is a multi-criteria decision making process from the perspective of safety[4]. A food safety risk evaluation index system is constructed based on the whole production process. This provides a convenient vehicle to assess food supplier choice, incorporating Food Safety laws and other the food quality management standards. The proposed index includes six first level indicators: personnel, production equipment, raw material, process, production environment, and management risk; and 15 secondary indicators. Following subsections will give an insight about these indicators, as shown in Table 1.

**Table 1:** Evaluation index for food safety risk.

Level 1:Indicator	Level 2: Indicator
Personnel	Professional and technical personnel ability
	Personnel food safety knowledge level
Production equipment	Equipment operation
	Equipment maintenance
Raw material	Testing of raw materials
	Traceability of raw materials
Methodological	Advanced production technology
	Continuous processing capacity
Production environment	Engineering infrastructure level
	Warehousing and security capabilities
Management	Production qualification
	Food quality assurance system
	Food inspection and testing capabilities
	Food safety education and training system
	Implementation of food safety system

### Personnel risk

Personnel risk mainly refers to the risk of food safety, which is caused by the food supplier in the process of food production and circulation due to human factors[21]. This involves the capability of professional technical personnel and the personnel’s knowledge about food safety. The former is the professional technical personnel’s control ability of food safety risk, while the latter is relevant knowledge that staff at all levels possess in the process of food production and circulation. This indicator is reflected in Table 1.

### Production equipment risk

Production equipment risk mainly refers to the risk of food safety, which is caused by food production enterprises in the production process, due to factors of production equipment[22]. This involves the real-time operation of production equipment and the daily maintenance of equipment. This indicator is shown in Table 1.

### Raw material risk

Raw material risk mainly refers to the risk of food safety, which is caused by the food supplier in the process of raw material purchase, due to the quality control ability of raw materials and other factors[23]. This involves the detection and test ability of raw materials, and the traceability of raw materials. This indicator is shown in Table 1.

### Methodological risk

Methodological risk refers to the risk of food safety, which is caused by the food supplier in the process of food production, due to factors of the production industry[24]. This involves the advancement of technology and continuous processing ability, guaranteeing food quality and safety. This indicator is shown in Table 1.

### Production environment risk

Risk of production environment refers to the risk of food safety, which is caused by the food supplier in the process of food production and circulation, due to the risk factor of production environment[25]. This involves the level of engineering infrastructure and warehousing guarantee capability, which guarantees food safety. This indicator is reflected in Table 1.

### Management risk

Management risk refers to the risk of food safety, which is caused by the food supplier in the process of food production and circulation, due to management factors[26–27]. This involves production qualification, food quality assurance system, food detection and test ability, education and training system of food safety, and the implementation of a food safety system, which can guarantee food safety. This indicator is shown in Table 1.

## Multiple attribute decision model

### Network relationship characteristics

The decision issue is not only a multiple attribute, but also has multiple agent participation [28–29], and this participants cooperate with each other. This cooperation between information descriptors should contain two aspects: the self-weight of different actors in the multiple cooperation [30], and the potential subject behavior in the cooperation network. However, existing research only considers actor weights in the cooperative network, i.e., the frequency of participation in the cooperation, and ignores subject behavior. This limitation leads to lack of comprehensiveness and consequential ineffective expression of the complexity of large datasets [31], and the relationship(s) between micro and macro behavior [32].

Recent research has started to consider the influence of differences between actors in the relation-network. Granovetter(1973),analyzed the strength of relationship between role nodes in the labor market[33]. Brass studied relationships and unethical behaviors for an actor network through structural relationships between the organization and the strength of the link between individuals [34]. Ghoshal introduced the individual network status based on role discovery [35]. Liu Xuan studied the influence of individual potential on knowledge diffusion in scientific research networks, and concluded that the individual potential measured by the center degree had a significant positive effect on knowledge diffusion [36], as shown in (S1 Fig).

### Multiple subject group language variable description

The description of language variables includes uncertainty [5], and an appropriate language assessment standard is required to obtain a qualitative measure of a subject’s language variables [6]. The five level language scale is ideal, but is often unable to express differences in the intuitive feelings of different actor-subject pairs. Therefore, a first order scale between adjacent levels of the five level scale was added, *T* = {*t*_1_,*t*_2_,*t*_3_,*t*_4_,*t*_5_,*t*_6_,*t*_7_,*t*_8_,*t*_9_}, where *t*_1_, *t*_3_, *t*_5_, *t*_7_, *t*_9_ are strongly do not agree, do not agree, agree in general, agree, strongly agree, respectively, *t*_2_, *t*_4_, *t*_6_, *t*_8_ are between *t*_1_ and *t*_3_, *t*_3_ and *t*_5_, *t*_5_ and *t*_7_, and *t*_7_ and *t*_9_, respectively, and 0.1 < *t*_*i*_ < 0.9, (*i* = 1,2,⋯,9). Once the nine-level scale is determined, using the exact number can lose the fuzzy information of fuzzy language. Therefore, a definition of the uncertainty is required.

Definition 1 [7]. Assume that *t* = [*t*_*a*_,*t*_*b*_], 0.1 < *t*_*a*_,…,*t*_*b*_ < 0.9, *t*_*a*_, *t*_*b*_ ∈ *T*, *a* ≤ *b*, $\tilde{t}$ are uncertain language variables.

Then, using the distance between uncertain language variables proposed by Newman [8].

Definition 2. Suppose ${\tilde{t}}_{1}=[t_{a1},t_{b1}]$, and ${\tilde{t}}_{2}=[t_{a2},t_{b2}]$ are uncertain language variables. Then the distance between ${\tilde{t}}_{1}$ and ${\tilde{t}}_{2}$ is

$$D({\tilde{t}}_{1},{\tilde{t}}_{2})=|\frac{\left(t_{a1}-t_{a2})+(t_{b1}-t_{b2}\right)}{2(t_{9}-t_{1})}|,$$

and $0\leq D({\tilde{t}}_{1},{\tilde{t}}_{2})\leq 1$.

From the theory of intuitive fuzzy sets [9], actor behaviors were divided into support, opposition, and neutrality. However, actor behavior is limited by their own knowledge and expression ability. Thus, support, opposition, and neutrality of language descriptions are often inconsistent [10], and choosing a single language is often not reasonable. Some behavior subjects, especially those that are vaguer about their own viewpoints and expressions, are able to draw more reasonable conclusions regarding the three options for language surveys and language variables.

Given the nine-level language scale, the support, oppose and neutrality sets are, respectively,

$$\begin{array}{c} T=\{t_{1}=0.1,t_{2}=0.2,t_{3}=0.3,t_{4}=0.4,t_{5}=0.5,\\ t_{6}=0.6,t_{7}=0.7,t_{8}=0.8,t_{9}=0.9\} \end{array};$$

$$\begin{array}{c} F=\{f_{1}=0.1,f_{2}=0.2,f_{3}=0.3,f_{4}=0.4,f_{5}=0.5,\\ f_{6}=0.6,f_{7}=0.7,f_{8}=0.8,f_{9}=0.9\} \end{array};$$

and

$$\begin{array}{c} Z=\{z_{1}=0.1,z_{2}=0.2,z_{3}=0.3,z_{4}=0.4,z_{5}=0.5,\\ z_{6}=0.6,z_{7}=0.7,z_{8}=0.8,z_{9}=0.9\} \end{array}.$$

The corresponding language variables are $\tilde{t}=[t_{a},t_{b}]$, $\tilde{f}=[f_{a},f_{b}]$, and $\tilde{z}=[z_{a},z_{b}]$, respectively, and the language logic is

$$t_{a}+f_{a}+z_{b}=1$$

and

$$t_{b}+f_{b}+z_{a}=1.$$

However, quantification of the behavioral language description cannot satisfy this logical relationship, due to inconsistency of the language description. To solve this discrepancy, language descriptions for the three attitudes can be standardized as

$${\bar{t}}_{a}=\frac{t_{a}}{t_{a}+f_{a}+z_{b}},\;{\bar{t}}_{b}=\frac{t_{b}}{t_{b}+f_{b}+z_{a}};$$

$${\bar{f}}_{a}=\frac{f_{a}}{t_{a}+f_{a}+z_{b}},\;{\bar{f}}_{b}=\frac{f_{b}}{t_{b}+f_{b}+z_{a}};$$

and

$${\bar{z}}_{a}=\frac{z_{a}}{t_{a}+f_{a}+z_{b}},\;{\bar{z}}_{b}=\frac{z_{b}}{t_{b}+f_{b}+z_{a}}.$$

Thus, the standardized language variables are $\bar{\tilde{t}}=[{\bar{t}}_{a},{\bar{t}}_{b}]$, $\bar{\tilde{f}}=[{\bar{f}}_{a},{\bar{f}}_{b}]$, and $\bar{\tilde{z}}=[{\bar{z}}_{a},{\bar{z}}_{b}]$, respectively.

### Interval intuitive fuzzy matrices

Definition 3 [8]. Let *X* be an intuitive fuzzy set, defined as:

$$A=\{<x,\mu_{A}(x),\upsilon_{A}(x)>|x\in X\},$$

where *μ*_*A*_(*x*): *X* → [0,1], *υ*_*A*_(*x*): *X* → [0,1], *μ*_*A*_(*x*) and *υ*_*A*_(*x*) are the membership and non-membership of *x* belonging to *A*, 0 ≤ *μ*_*A*_(*x*) + *υ*_*A*_(*x*) ≤ 1, *x* ∈ *X*.

Definition 4 [9]. For some set *X*, the interval value on the intuitive fuzzy set is defined as

$$A=\{<x,[\mu_{AL}(x),\mu_{AU}(x)],[\upsilon_{AL}(x),\upsilon_{AU}(x)]>|x\in X\},$$

Where 0 ≤ *μ*_*AU*_(*x*) + *υ*_*AU*_(*x*) ≤ 1, *μ*_*AL*_ ≥ 0, and *υ*_*AL*_(*x*) ≥ 0; and *x* is the interval value of the set *A*. The intuitive fuzzy hesitation is

$$\pi_{A}(x)=[1-\mu_{AU}(x)-\upsilon_{AU}(x),1-\mu_{AL}(x)-\upsilon_{AL}(x)].$$

Thus, if the research group is domain *X*, then one of the actors in the group is *x*, and the corresponding language variables are standardized as the interval intuitive fuzzy sets $A=\{<x,{\bar{\tilde{t}}}_{i}={\left[{\bar{t}}_{a},{\bar{t}}_{b}\right]}_{x},{\bar{\tilde{f}}}_{i}={\left[{\bar{f}}_{a},{\bar{f}}_{b}\right]}_{x}>|x\in X\}$. The behavior subject, *x*, corresponds to a three-dimensional interval vector, ${\left({\left[{\bar{t}}_{a},{\bar{t}}_{b}\right]}_{x},{\left[{\bar{f}}_{a},{\bar{f}}_{b}\right]}_{x},{\left[{\bar{z}}_{a},{\bar{z}}_{b}\right]}_{x}\right)}^{T}$, in *A*, and a group interval intuitive fuzzy matrix can be formed based on *A*.

Definition 5[11]. Let *X* be some a group as defined above, and |*X*| = *N*. Then the ordered element of *X* is {*x*_1_,*x*_2_,⋯,*x*_*N*_}, and *A* corresponds to a unique matrix, *M*_*A*_, on *X*,

$$M_{A}={\left(m_{ij}\right)}_{3\times N}=\left(\begin{array}{llll} {\left[{\bar{t}}_{a},{\bar{t}}_{b}\right]}_{x_{1}} & {\left[{\bar{t}}_{a},{\bar{t}}_{b}\right]}_{x_{2}} & \cdots & {\left[{\bar{t}}_{a},{\bar{t}}_{b}\right]}_{x_{N}}\\ {\left[{\bar{f}}_{a},{\bar{f}}_{b}\right]}_{x_{1}} & {\left[{\bar{f}}_{a},{\bar{f}}_{b}\right]}_{x_{2}} & \cdots & {\left[{\bar{f}}_{a},{\bar{f}}_{b}\right]}_{x_{N}}\\ {\left[{\bar{z}}_{a},{\bar{z}}_{b}\right]}_{x_{1}} & {\left[{\bar{z}}_{a},{\bar{z}}_{b}\right]}_{x_{2}} & \cdots & {\left[{\bar{z}}_{a},{\bar{z}}_{b}\right]}_{x_{N}} \end{array}\right),$$

Where *M*_*A*_ is called the interval intuitive fuzzy matrices on group *X*.

### Evaluation of individual potential based on network relationship

For the relationship between decision-making groups, network topology is represented by a quaternion *G* = <*V*,*E*,*ϕ*(*V*),*φ*(*E*)>, where *V* indicates the set of nodes, i.e., the set of behavioral subjects, and |*V*| = *n* indicates that *n* has a node in the network topology; *E* (|*E*| ≤ *n*(*n*−1)) indicates the edge set associated with the node, and is a collection of cooperative relationships between actors; *ϕ*(*V*) is a collection of all nodes participating in the collaborations, and *ϕ*(*v*_*i*_) is abbreviated as *ϕ*_*i*_; and *φ*(*E*) is the composition number of cooperation on the edge of the set, to determine whether there is an edge and measure the thickness. If there is no cooperative relationship between nodes *v*_*i*_ and *v*_*j*_, then *φ*(*v*_*i*_,*v*_*j*_) = 0, which is abbreviated as *φ*_*ij*_ = 0.

Theorem 1. The number of times a node participates in the collaborations is equal to the sum of the number of collaborations associated with all edges, i.e., $\phi_{i}=\sum_{j=1}^{n}\varphi_{ij}$, *i* = 1,⋯,*n*.

This is self-evident, and does not require proof.

Definition 6. The cooperative relationship in the network topology is represented by the cooperation matrix *H* = (*φ*_*ij*_)_*n*×*n*_.

Definition 7 [12]. Let *N*(*A*) ≡ ‖*A*‖ be a non-negative real valued function of matrix *A* ∈ *R*^*n*×*m*^. Then, for any *n*×*m* matrixes *A* and *B*, the following conditions are met.

1. Positive qualitative: ‖*A*‖ ≥ 0, and ‖*A*‖ = 0 ⇔ *A* = 0.
2. Homogeneous: ‖*αA*‖ = |*α*|‖*A*‖, *α* ∈ *R*.
3. Triangular inequality: for any two matrixes *A* and *B* of the same type ‖*A* + *B*‖ ≤ ‖*A*‖ + ‖*B*‖.
4. Matrix multiplication compatibility: If *A* and *B* can be multiplied, then ‖*AB*‖ ≤ ‖*A*‖‖*B*‖.

Then *N*(*A*) is a matrix norm on *R*^*n*×*m*^, and the Frobenius expression is $\left\|A\right\|=(\sum_{i=1}^{n}\sum_{j=1}^{m}a_{ij}{}^{2}{)}^{1/2}$.

### Multiple attribute decision model

Suppose *A* = {*A*_1_,*A*_2_,⋯,*A*_*m*_} is a set of scenarios with *G* = {*G*_1_,*G*_2_,⋯,*G*_*n*_} a decision making group, where *G*_*i*_ on the degree of recognition of *A*_*i*_ corresponds to language standardization formation of a three-dimensional interval vector $m_{ij}={\left({\left[\bar{t_{a}},\bar{t_{b}}\right]}_{A_{i}},{\left[\bar{f_{a}},\bar{f_{b}}\right]}_{A_{i}},{\left[\bar{z_{a}},\bar{z_{b}}\right]}_{A_{i}}\right)}^{T}$. Then the degree of recognition of scheme *A*_*i*_ by a decision maker is *M*_*A*_ = (*m*_*ij*_)_3×*n*_. The cooperative relationship in the network relationship topology in the decision making community is the cooperation matrix, *H* = (*φ*_*ij*_)_*n*×*n*_, where *φ*_*ij*_ is the number of collaborations between the two decision makers. By comparing condition number (cond(A) = ‖*A*‖∙‖*A*^−1^‖) of the matrix in all solutions, and using it to multiply by the weight, and the smaller the resulting value is, the better the solution will be. Thus we can determine the best solution through this method.

*D* = {*D*_1_,*D*_2_,⋯,*D*_*p*_} is the attribute set, and its weight is *γ*_*i*_, which conforms to *γ*_*i*_ ∈ [0,1], $\sum_{i=1}^{p}\gamma_{i}=1$; the attribute value is ${\tilde{x}}_{ij}=<[{\bar{t}}_{aij},{\bar{t}}_{bij}],[{\bar{f}}_{aij},{\bar{f}}_{bij}],[{\bar{z}}_{aij},{\bar{z}}_{bij}]>$, *i* = 1,2,⋯,*m*, and *j* = 1,2,⋯,*n*, and the decision making matrix is $\tilde{X}={\left[{\tilde{x}}_{ij}\right]}_{m\times n}={\left([{\bar{t}}_{aij},{\bar{t}}_{bij}],[{\bar{f}}_{aij},{\bar{f}}_{bij}],[{\bar{z}}_{aij},{\bar{z}}_{bij}]\right)}_{m\times n}$. *G* = {*G*_1_,*G*_2_,⋯,*G*_*n*_} is the decision making group, in which *w*_*i*_ refers to the weight of attribute *G*_*i*_, which conforms to 0 ≤ *w*_*i*_ ≤ 1, $\sum_{i=1}^{n}w_{i}=1$.

Definition 8:In decision making matrix with intuitive uncertain language information in the interval $\tilde{X}={\left[{\tilde{x}}_{ij}\right]}_{m\times n}={\left([{\bar{t}}_{aij},{\bar{t}}_{bij}],[{\bar{f}}_{aij},{\bar{f}}_{bij}],[{\bar{z}}_{aij},{\bar{z}}_{bij}]\right)}_{m\times n}$, assume *A*_*i*_(*w*) is the deviation between solutions *A*_*i*_ and *A*_*q*_, then:

$$\begin{array}{l} A_{i}(w)=\sum_{j=1}^{n}\sum_{q=1}^{m}d_{NH}({\tilde{x}}_{ij},{\tilde{x}}_{qj})w_{j}\\ =\frac{1}{8(l-1)}\sum_{j=1}^{n}\sum_{q=1}^{m}\left[\begin{array}{l} \left|\left(1+{\bar{f}}_{aij}-{\bar{z}}_{aij})(a_{ij}+b_{ij})-(1+{\bar{f}}_{aqj}-{\bar{z}}_{aij})(a_{qj}+b_{qj}\right)\right|+\\ \left|\left(1+{\bar{f}}_{bij}-{\bar{z}}_{bij})(a_{ij}+b_{ij})-(1+{\bar{f}}_{bij}-{\bar{z}}_{bij})(a_{qj}+b_{qj}\right)\right| \end{array}\right]w_{j} \end{array}$$

Where: *i* = 1,2,⋯,*m*.

Solve the optimal solution of weight and carry out unification processing, we can obtain that:

$$w_{j}=\frac{\sum_{i-1}^{m}\sum_{q=1}^{m}\left[\begin{array}{l} \left|\left(1+{\bar{f}}_{aij}-{\bar{z}}_{aij})(a_{ij}+b_{ij})-(1+{\bar{f}}_{aqj}-{\bar{z}}_{aqj})(a_{qj}+b_{qj}\right)\right|+\\ \left|\left(1+{\bar{f}}_{bij}-{\bar{z}}_{bij})(a_{ij}+b_{ij})-(1+{\bar{f}}_{bqj}-{\bar{z}}_{bqj})(a_{qj}+b_{qj}\right)\right| \end{array}\right]}{\sum_{j=1}^{n}\sum_{i=1}^{m}\sum_{q=1}^{m}\left[\begin{array}{l} \left|\left(1+{\bar{f}}_{aij}-{\bar{z}}_{aij})(a_{ij}+b_{ij})-(1+{\bar{f}}_{aqj}-{\bar{z}}_{aqj})(a_{qj}+b_{qj}\right)\right|+\\ \left|\left(1+{\bar{f}}_{aij}-{\bar{z}}_{aij})(a_{ij}+b_{ij})-(1+{\bar{f}}_{bqj}-{\bar{z}}_{bqj})(a_{qj}+b_{qj}\right)\right| \end{array}\right]},j=1,2,\ldots ,n.$$

The weight of the decision making program is *W* = (*w*_*A*_ *w*_*B*_ *w*_*C*_)

The specific decision steps are as follows.

**Step 1**: *G*_*i*_ is the matrix norm of each scheme’s interval intuitive fuzzy matrix, hence *G*_*i*_ = *M*_*i*_ × *H*.
**Step 2**: From Definition 8, $\left\|A\right\|=(\sum_{i=1}^{n}\sum_{j=1}^{m}a_{ij}{}^{2}{)}^{1/2}$, The matrix norm of matrix *G*_*i*_ and the matrix norm of its inverse matrix can be obtained;
**Step 3**: To obtain the best solution, we calculate the number of conditions for each scheme matrix, *G*_*i*_;
**Step 4**: And we calculate *R* = (*r*_*i*_) = (*cond*(*A*) *cond*(*B*) *cond*(*C*))×*W*_*j*_ to get the final result and sort the scheme, where smaller *r*_*i*_ implies a superior scheme.

## Case study

Suppose a company, M, is a retail chain owned by a large supermarket chain with many suppliers, and need to purchase a batch of food. Up to five branch stores in multiple locations make purchasing decisions together, and the supermarket has three suppliers (A, B, C) who provide the particular product under consideration. Each branch director provides a food safety risk assessment for the three suppliers. Since the decision makers belong to different departments during the purchasing process, so the frequency of participation and the locations of cooperation are different. Therefore, their choice decision matrixes are Table 2.

**Table 2:** The choice decision matrix of supplier (A, B, C).

**Supplier A**	**Opposition**	**Support**	**Neutrality**
1	[0.8,0.9]	[0.1,0.1]	[0.2,0.3]
2	[0.6,0.9]	[0.2,0.2]	[0.3,0.5]
3	[0.8,0.9]	[0.1,0.1]	[0.1,0.2]
4	[0.8,0.8]	[0.1,0.2]	[0.2,0.2]
5	[0.7,0.9]	[0.2,0.3]	[0.2,0.3]
**Supplier B**	**Opposition**	**Support**	**Neutrality**
1	[0.7,0.8]	[0.2,0.3]	[0.3,0.5]
2	[0.8,0.9]	[0.1,0.2]	[0.2,0.3]
3	[0.7,0.9]	[0.1,0.2]	[0.2,0.4]
4	[0.7,0.8]	[0.1,0.1]	[0.3,0.3]
5	[0.6,0.8]	[0.1,0.1]	[0.2,0.5]
**Supplier C**	**Opposition**	**Support**	**Neutrality**
1	[0.7,0.9]	[0.2,0.2]	[0.2,0.3]
2	[0.7,0.7]	[0.1,0.2]	[0.1,0.3]
3	[0.6,0.9]	[0.1,0.3]	[0.2,0.5]
4	[0.8,0.8]	[0.1,0.2]	[0.2,0.3]
5	[0.7,0.8]	[0.1,0.1]	[0.2,0.4]

And the cooperative matrix representing the network relationship is

$$H=\left(\begin{array}{ccccc} 0 & 5 & 3 & 20 & 2\\ 5 & 0 & 8 & 5 & 0\\ 3 & 5 & 0 & 0 & 2\\ 20 & 0 & 0 & 0 & 0\\ 2 & 8 & 2 & 0 & 0 \end{array}\right).$$

From 3.5,Weight of scenarios is got as *W* = (0.27 0.31 0.42)

**Step 1:** The intuitive fuzzy matrices, *M*_*A*_,*M*_*B*_,*M*_*C*_, for each supplier interval are obtained and the cross-product matrices, *G*_*A*_,*G*_*B*_,*G*_*C*_ calculated for the cooperative matrix *H*,
  $$M_{A}=\left[\begin{array}{ccccc} 0.1 & 0.2 & 0.1 & 0.15 & 0.25\\ 0.85 & 0.75 & 0.85 & 0.8 & 0.8\\ 0.25 & 0.4 & 0.15 & 0.2 & 0.25 \end{array}\right],$$
  $$G_{A}=M_{A}\times H=\left[\begin{array}{ccccc} 4.8 & 3 & 2.4 & 3 & 0.4\\ 23.9 & 14.9 & 10.15 & 20.75 & 0.8\\ 6.95 & 4 & 4.45 & 7 & 0.8 \end{array}\right],$$
  $$G_{A}^{-}=\left[\begin{array}{ccc} 0.3 & 0.02 & -0.19\\ 0.19 & 0.05 & -0.26\\ 0.41 & -0.20 & 0.43\\ -0.69 & 0.09 & 0.17\\ 0.20 & -0.1 & 0.19 \end{array}\right];$$
  $$M_{B}=\left[\begin{array}{ccccc} 0.25 & 0.15 & 0.15 & 0.1 & 0.1\\ 0.75 & 0.85 & 0.8 & 0.75 & 0.7\\ 0.4 & 0.25 & 0.3 & 0.3 & 0.35 \end{array}\right],$$
  $$G_{B}=M_{B}\times H=\left[\begin{array}{ccccc} 3.4 & 2.8 & 2.15 & 5.75 & 0.8\\ 23.05 & 13.35 & 10.45 & 19.25 & 3.1\\ 8.85 & 6.3 & 3.9 & 9.25 & 1.4 \end{array}\right],$$
  $$G_{B}^{-}=\left[\begin{array}{ccc} -0.23 & 0.11 & -0.09\\ -0.6 & -0.26 & 0.92\\ 0.51 & 0.25 & -0.81\\ 0.4 & -0.03 & -0.1\\ 0.05 & 0.004 & -0.03 \end{array}\right];$$
  $$M_{C}=\left[\begin{array}{ccccc} 0.2 & 0.15 & 0.2 & 0.15 & 0.1\\ 0.8 & 0.7 & 0.75 & 0.8 & 0.75\\ 0.25 & 0.2 & 0.35 & 0.25 & 0.3 \end{array}\right],$$
  $$G_{C}=M_{C}\times H=\left[\begin{array}{ccccc} 4.55 & 2.8 & 2 & 4.75 & 0.8\\ 23.25 & 13.75 & 9.5 & 19.5 & 3.1\\ 7.65 & 5.4 & 2.95 & 6 & 1.2 \end{array}\right],$$
  and
  $$G_{C}^{-}=\left[\begin{array}{ccc} -0.98 & 0.36 & -0.38\\ 0.33 & -0.38 & 0.96\\ -0.22 & 0.14 & -0.27\\ 0.97 & -0.15 & -0.14\\ 0.44 & -0.2 & 0.32 \end{array}\right].$$
**Step 2**: Since $\left\|A\right\|=(\sum_{i=1}^{n}\sum_{j=1}^{m}a_{ij}{}^{2}{)}^{1/2}$, the Frobenius matrix norms of $G_{A},G_{A}^{-},G_{B},G_{B}^{-},G_{C},G_{C}^{-}$ are
  $$\left\|G_{A}\right\|=38.83,$$
  $$\left\|G_{A}^{-}\right\|=1.11,$$
  $$\left\|G_{B}\right\|=38.44,$$
  $$\left\|G_{B}^{-}\right\|=1.58,$$
  $$\left\|G_{C}\right\|=37.41,$$
  and
  $$\left\|G_{C}^{-}\right\|=1.97.$$
**Step 3**: The condition number for each supplier is calculated as
  $$cond(A)=\left\|G_{A}\right\|\left\|G_{A}^{-}\right\|=43.1,$$
  $$cond(B)=\left\|G_{B}\right\|\left\|G_{B}^{-}\right\|=60.74,$$
  and
  $$cond(C)=\left\|G_{C}\right\|\left\|G_{C}^{-}\right\|=73.7.$$
Step 4: We get *R* = (11.64 18.83 30.95).

Finally, we compare the size of the value and show that *R*_*A*_ < *R*_*B*_ < *R*_*C*_. Since smaller condition value implies superior food safety and quality for the corresponding supplier, in this case the A supplier would be preferred.

## Conclusions

The relationship between decision making characteristics was analyzed and a new network multiple attribute decision making method based on IVIFS characteristics was proposed. A case study showed how the proposed model was applied to food safety risk. Specificity of supermarket food purchasing decisions were analyzed, and factors influencing index selection were identified. A supplier evaluation index system was determined based on the food production process, and then an interval intuitive fuzzy set evaluation model was established using the proposed method. Smaller final condition score values indicated superior suppliers, and so the minimum condition was chosen, corresponding to the specific supplier. Thus, the multiple attribute decision making problem for supermarket food supplier selection was solved using the proposed model, which verified that the model accurately and adaptively solved the multiple attribute decision making problem.

## Supporting information

S1 Fig Language scale. (TIF)

S1 File Editing professional service. (PDF)
