# Supplementary figures and images for: Multiple attribute decision making model and application to food safety risk evaluation
Source: PLoS One. 2017 Dec 19;12(12):e0189835. doi: 10.1371/journal.pone.0189835 (PMC5736234; doi:10.1371/journal.pone.0189835)

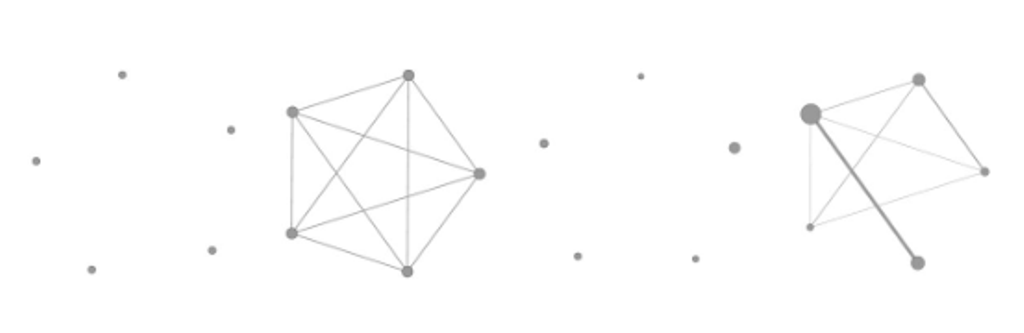

Supplement: S1 Fig — (TIF) [file pone.0189835.s001.tif]
