# Supplementary material for: Multiple attribute decision making model and application to food safety risk evaluation
Source: PLoS One. 2017 Dec 19;12(12):e0189835. doi: 10.1371/journal.pone.0189835 (PMC5736234; doi:10.1371/journal.pone.0189835)
Supplement: S1 File — (PDF) [file pone.0189835.s002.pdf]

Editing professional service

The revision is edited by CTIS(Beijing Chinese-Foreign Translation & Information Service Co., Ltd.)
